# New descriptions of the larval and pupal stages of *Orthocladiusnitidoscutellatus* and *Psectrocladiusnevalis* from Xizang, China (Diptera, Chironomidae)

**DOI:** 10.3897/BDJ.12.e121952

**Published:** 2024-04-03

**Authors:** Xinyu Ge, Chengyan Wang, Wenxuan Pei, Yaning Tang, Wenbin Liu, Chuncai Yan

**Affiliations:** 1 Tianjin Key Laboratory of Conservation and Utilization of Animal Diversity, Tianjin Normal University, Tianjin, China Tianjin Key Laboratory of Conservation and Utilization of Animal Diversity, Tianjin Normal University Tianjin China

**Keywords:** Chironomidae, *Orthocladius*, *Psectrocladius*, larval, pupal, Tibetan Plateau

## Abstract

**Background:**

Tibetan Plateau is one of the most typical areas of biodiversity in the world because of its unique environmental and regional units, which breed unique biological communities and concentrate on many unique and rare wild animals and plants. Research on Chironomidae in the Tibetan Plateau is relatively weak. At present, the identification of Chironomidae species mainly depends on male adults, while identification of larvae and pupae is relatively difficult and there is less research on them.

**New information:**

During the investigations of insect diversity in the Tibetan Plateau, larval and pupal stages of *Orthocladiusnitidoscutellatus* Lundström, 1915 and *Psectrocladiusnevalis* Akhrorov, 1977 were described and illustrated. Matching and identification of larval and pupal stages were based on DNA barcodes. Neighbour-joining trees were reconstructed, based on known *Orthocladius* and *Psectrocladius* COI DNA barcodes, respectively.

## Introduction

Chironomidae is one of the most diverse and abundant groups of insects found in various habitats in global aquatic ecosystems, widely used for the impact of pollutants in the aquatic system (Liu et al. 2022). Even for the experienced observer, the aquatic larval level and pupal stages are quite similar and almost impossible to be separated, based purely on morphological criteria (Armitage et al. 1995, Rossaro et al. 2022). The DNA barcodes corresponding to the 658-bp fragment of the mitochondrial gene cytochrome c oxidase I (COI) has been identified as the core of a global bioidentifcation system at the species level and proved to be useful in the delimitation of non-biting midge species (Ashe and O'Connor 2012, Gilka et al. 2018, Liu et al. 2021, Liu et al. 2023, Hebert et al. 2003a, Hebert et al. 2003b).

The genus *Orthocladius* Wulp, 1874, includes 144 species worldwide and is one of the richest in species within the Chironomidae subfamily Orthocladiinae (Ashe and O'Connor 2012, Moubayed et al. 2022, Rossaro et al. 2022). *Orthocladius* larvae and pupae are rather similar in morphology (Langton and Visser 2003,Epler et al. 2013). Most species live in running waters like rivers and streams, while a few can be found in standing waters, such as ditches and lakes (Cuppen and Tempelman 2022). *Orthocladiusnitidoscutellatus* Lundström, 1915 was reported in east Siberia and described, based on adult males (Lundström 1915). It widely distributed in some countries of the Palearctic and Nearctic Regions, but there is currently no detailed description of larvae and pupae (Ashe and O'Connor 2012, Rossaro et al. 2022).

Kieffer (1906) erected the genus *Psectrocladius* with *Orthocladiuspsilopterus* Kieffer, 1906 as the type species. This genus is divided into four subgenera, with a current world record of 61 valid species (Ashe and O'Connor 2012). Since the first report of the male adults of *Psectrocladiusnevalis* Akhrorov, 1977 in Lake Zorkul on the Pamir Plateau in Tajikistan, there has been little research related to this species (Akhrorov 1977,Ashe and O'Connor 2012). To date, only simple descriptions of male adults of this species and public data about its barcode by Chinese scholars are available (Han et al. 2023).

The Tibetan Plateau is one of the most important areas of biodiversity in the world because of its unique environmental and regional units, which breed unique biological communities and many unique and rare wild animals and plants (Liu et al. 2023). Here, we described and illustrated larval and pupal stages of *Orthocladiusnitidoscutellatus* Lundström, 1915 and *Psectrocladiusnevalis* Akhrorov, 1977 from the Tibetan Plateau. Matching and identification of larval and pupal stages are based on DNA barcodes. Neighbour-joining trees were reconstructed, based on known *Orthocladius* and *Psectrocladius* COI DNA barcodes, respectively.

## Materials and methods

The examined specimens were caught using sweepnets and light traps, stored in the dark at 4^0^C, and preserved in 85% ethanol before molecular and morphological analyses. Genomic DNA was extracted from the thorax and leg using a Qiagen DNA Blood and Tissue Kit at Tianjin Normal University, Tianjin, China (**TJNU**), following the standard protocol, except for the final elution volume of 100 µl. After DNA extraction, the exoskeleton of each specimen was mounted in Euparal on a microscope slide together with the corresponding antennae, legs, wing and abdomen, following the procedures outlined by Sæther (1980). Morphological terminology follows Sæther (1969).

The colour pattern of all species is described, based on the specimen preserved in ethanol. Digital photographs of slide-mounted specimens were taken with a 300-dpi resolution using Nikon Eclipse 80i with Nikon Digital Sight DS-Fil camera at TJNU.

The universal primers LCO1490 and HCO2198 (Folmer et al. 1994) were adopted to amplify the standard 658-bp mitochondrial COI barcode region. Polymerase chain reaction (PCR) amplifications followed Song et al. (2018) and were conducted in a 25 µl volume including 12.5 µl 2× Es Taq MasterMix (CoWin Biotech Co., Beijing, China), 0.625 µl of each primer, 2 µl of template DNA and 9.25 µl of deionised H_2_O. PCR products were electrophoresed in 1.0% agarose gel, purified and sequenced in both directions at Beijing Genomics Institute Co. Ltd., Beijing, China.

Raw sequences were assembled and edited in Geneious Prime 2020 (Biomatters Ltd., Auckland, New Zealand). Alignment of the sequences was carried out using the MUSCLE (Edgar 2004) algorithm on amino acids in MEGA v. 7.0 (Kumar et al. 2016). Some published DNA barcodes were downloaded from the Barcode of Life Data Systems (BOLD) (Ratnasingham and Hebert 2013). Before phylogenetic analysis, nucleotide substitution saturation analysis of gene sequences was performed by DAMBE version 6 (Xia 2017). The pairwise distances were calculated using the Kimura 2-Parameter (K2P) substitution model in MEGA. The Neighbour-joining (NJ) tree was constructed using the K2P substitution model, 1000 bootstrap replicates and the “pairwise deletion” option for missing data in MEGA. Novel sequences, trace-files and metadata of the new species were uploaded to the BOLD platform.

In this study, the partial COI sequences of *Parachironomus* were submitted to online ABGD web interface (https://bioinfo.mnhn.fr/abi/public/abgd/abgdweb.html). We used the K2P nucleotide substitution model. The prior intraspecific divergence was set at between 0.001 and 0.1. The minimum relative gap width was 1.0 and other parameters were defaulted.

## Taxon treatments

### Orthocladius nitidoscutellatus

Lundström, 1915

3FF18C20-4F95-5C3A-8BC8-8A71AFDEC555

*Orthocladiusnitidoscutellatus* Lundström, 1915- Lundström (1915):11
*Orthocladiustrigonolabis* Edwards, 1924 - Edwards (1924): 170
*Orthocladiusaquilonaris* Goetghebuer, 1940 - Goetghebuer (1940): 63
*Orthocladiusnitidoscutellatus* Lundström, 1915 - Sæther (2004): 14; Ashe and O'Connor (2012): 434; Rossaro et al. (2022): 8

#### Materials

- **Type status:** Other material. **Occurrence:** lifeStage: 1 Larva, 1 Pupa; occurrenceID: B6A08146-047E-5C20-8B5C-A354B2AED520; **Location:** country: China; stateProvince: Xizang Autonomous Region; locality: Naqu City, Sena District, Naqu Bridge on the Naqu River,; verbatimLatitude: 31°42.78′N; verbatimLongitude: 91°98.82′E; **Event:** eventDate: 7 Jul 2022; **Record Level:** institutionCode: Tianjin Normal University, Tianjin, China (TJNU)

#### Description

**Pupa (n = 1).**

**Figure 1.**
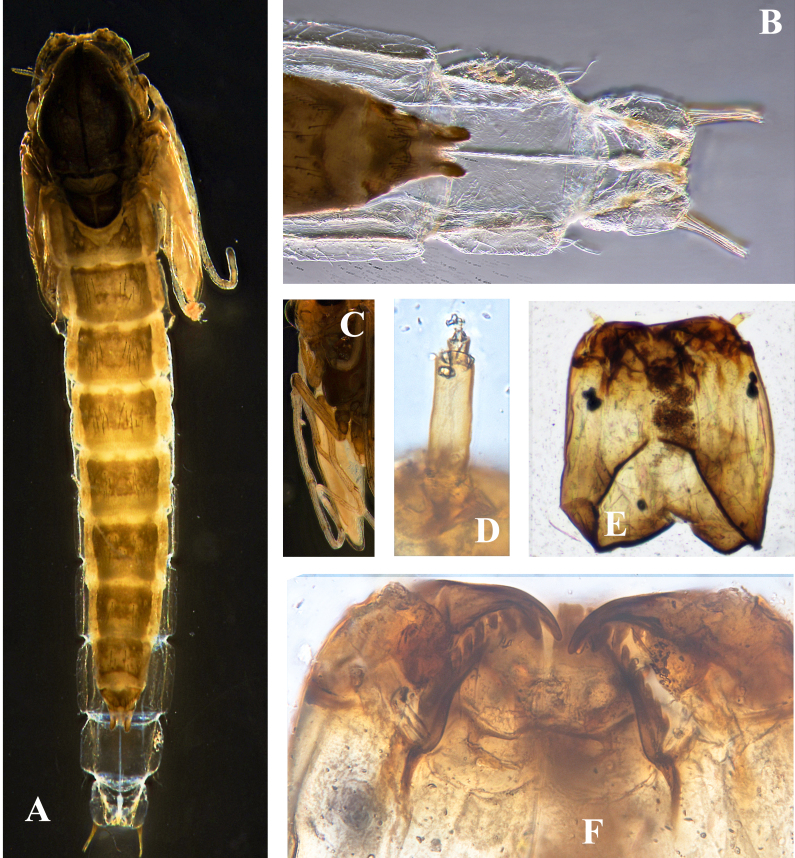
*Orthocladiusnitidoscutellatus* Lundström, 1915. **A** pharate adult with pupae exuviae; **B** sternites VIII–IX; **C** wing sheath; **D** antenna; **E** Larval head shell; **F** mentum and mandible.

**Figure 2.**
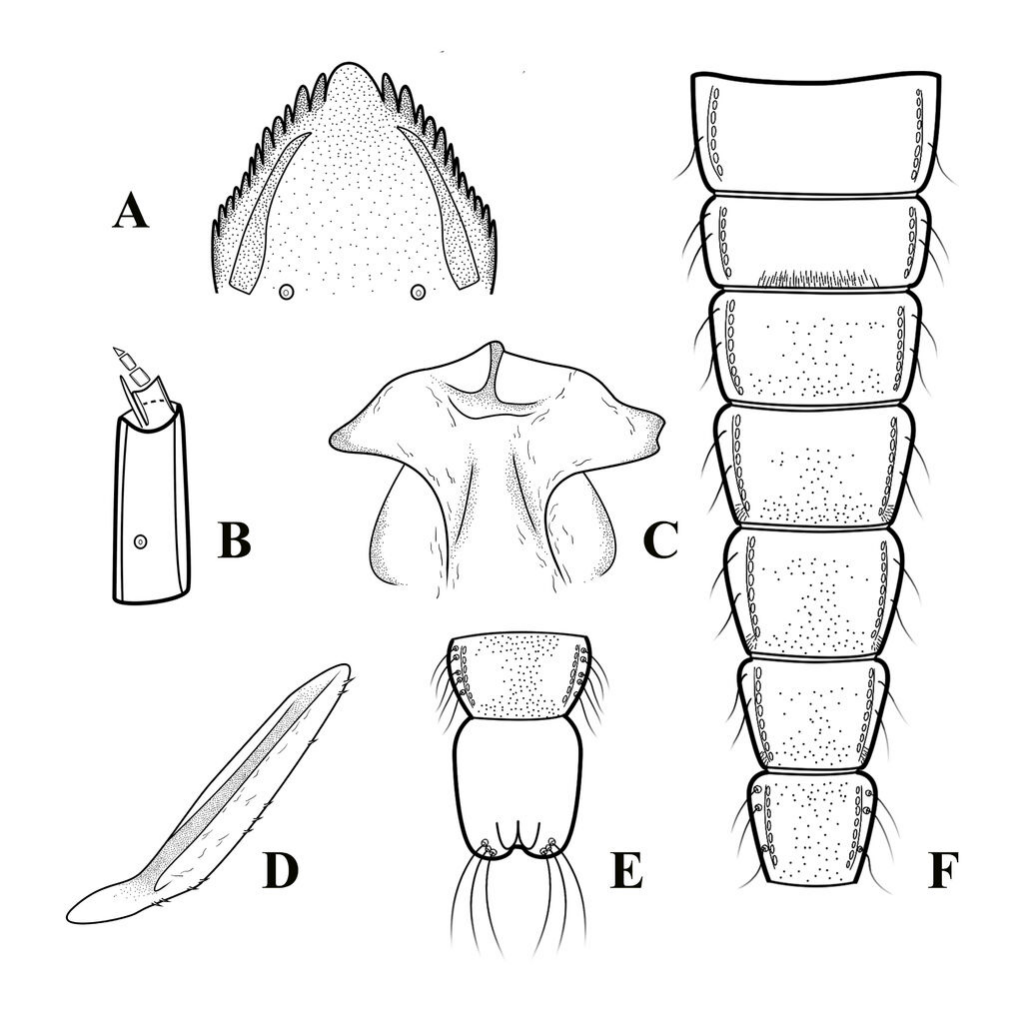
*Orthocladiusnitidoscutellatus* Lundström, 1915. **A** mentum; **B** antenna; **C** frontal apotome; **D** thoracic horn; **E** sternites VIII–IX; **F** segment I-VII.

Cephalothorax (Fig. 1C, Fig. 2 C-D). Cephalic tubercles and frontal setae absent. Thoracic horn elongated, 429.62 μm long, 35.25 μm wide. Thorax smooth, free of tubercles. Wing sheath without protrusions. Next to the thoracic horn, three precorneal setae are present, lengths of precorneals (µm): 32.50, 72.40, 91.10. Four dorsocentral setae, lengths of dorsocentrals (µm): 120.18, 145.20, 150.25, 48.50.

Abdomen (Fig. 1A-B, Fig. 2E-F). Sternites I– II bare; Posterior row of hooks, extending nearly on 2/3 the width of tergite II; Sternites III–VI with posteromedian shagreen composed of large spines; Sternites VII–VII and anterior row of anal lobe with shagreen composed of fine spines. Pedes spurii A present in segment IV–VI. Pedes spurii B present on segment II. These are teeth at the distal margin of the anal macrosetae. Tergite I with 1 Lt-setae; tergites II–VI with 2 Lt-setae. tergite VII with 3 Lt-setae. Tergite VIII with 5 Lt-setae. Anal lobe 240.74 µm long and 246 µm wide, with 3 subequal anal macrosetae. Anal lobe genital sheath 111.12 µm long, non-extending anal lobe.

**Fourth instar larva** (n = 1).

Head capsule (Fig. 1E) length / width: 294.69 µm / 304.13 µm: 0.97.

Colouration. Head capsule brown, mentum and the apical part of mandible dark brown; posterior occipital margin brown. Antenna (Fig. 1D, Fig. 2B) with each segments length (μm): 43.06, 11.45, 5.55, 4.46, 2.65. AR 1.79. Basal segment 16.59 μm wide, the basal segment length 2.60 times the width; the distance from base to ring organ 15.3 μm. Premandible 51.14 μm long, terminating in a single tooth. Mandible (Fig. 1F, Fig. 2A) 122 μm long, with 1 apical and 3 visible inner teeth, apical tooth 14.29 μm long, three inner teeth 23.74 μm wide; the outer margin smooth. Mentum (Fig. 1F, Fig. 2A) 1 median tooth and 12 pairs of lateral teeth, median tooth 21.65 μm wide, about 3.27 times larger than the first lateral tooth; mentum 113.3 μm wide; postmentum 130.1 μm long; the distance between the setae submenti 42.41 μm, the setae submenti slightly behind mentum. Abdomen have 4 anal tubules: 2 dorsal and 2 ventral ones. Segments I-VIII are rather similar in shape being quite symmetrical. Segment IX has the dorsal margin longer than the ventral margin, the dorsal margin carrying the procercus. Segment X is short, the posterior pseudopods being attached to its distal margin.

### Psectrocladius nevalis

Akhrorov, 1977

7F8062F2-06F9-57BC-B52B-09881B3D7C9F

*Psectrocladiusnevalis* Akhrorov, 1977 - Akhrorov (1977): 14; Ashe and O'Connor (2012): 516

#### Materials

- **Type status:** Other material. **Occurrence:** lifeStage: 2 Larvae I 1 Pupa; occurrenceID: 9B86C70A-908A-5E53-B595-F6F14D98C454; **Location:** country: China; stateProvince: Xizang Autonomous Region; locality: Naqu City, Sena District, Naqu Bridge on the Naqu River; verbatimLatitude: 31°42.78′N; verbatimLongitude: 91°98.82′E; **Event:** eventDate: 7 Jul 2022; **Record Level:** institutionCode: Tianjin Normal University, Tianjin, China (TJNU)

#### Description

**Pupa** (n = 1).

**Figure 3.**
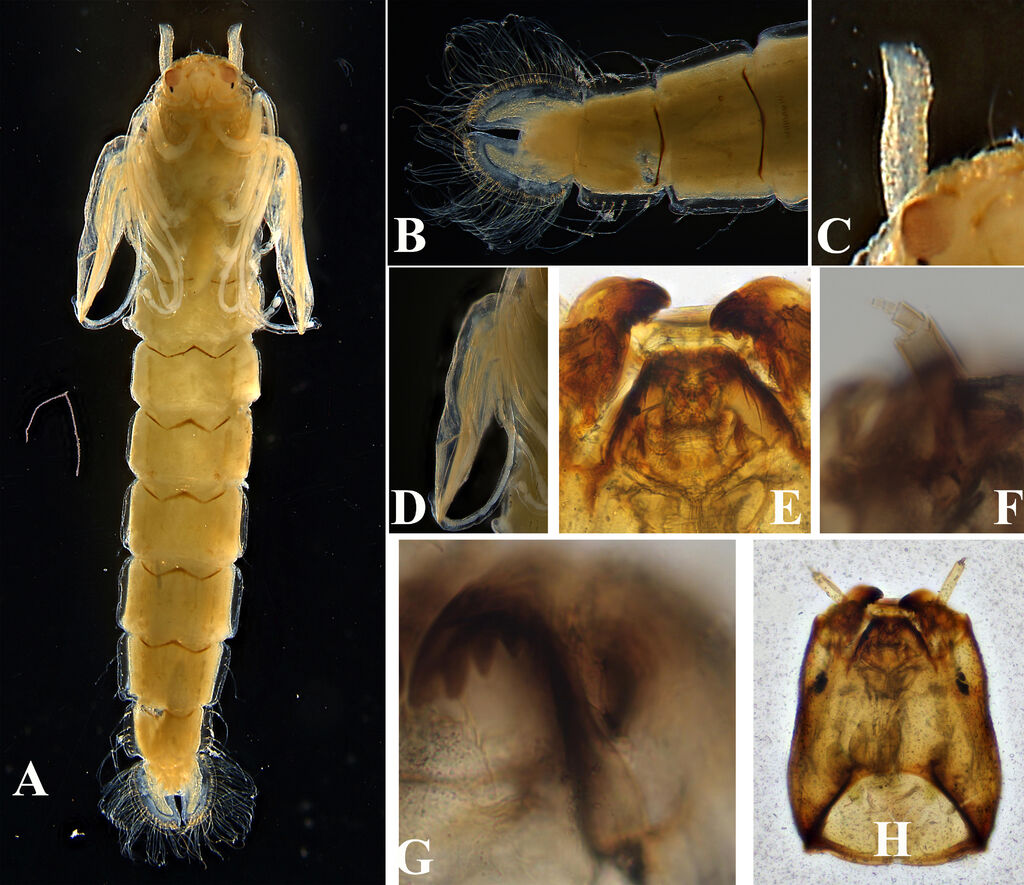
*Psectrocladiusnevalis* Akhrorov, 1977. **A** pharate adult with pupae exuviae; **B** sternites VIII–IX; **C** thoracic horn; **D** wing sheath; **E** mentum; **G** mandible; **H** larval head shell.

**Figure 4.**
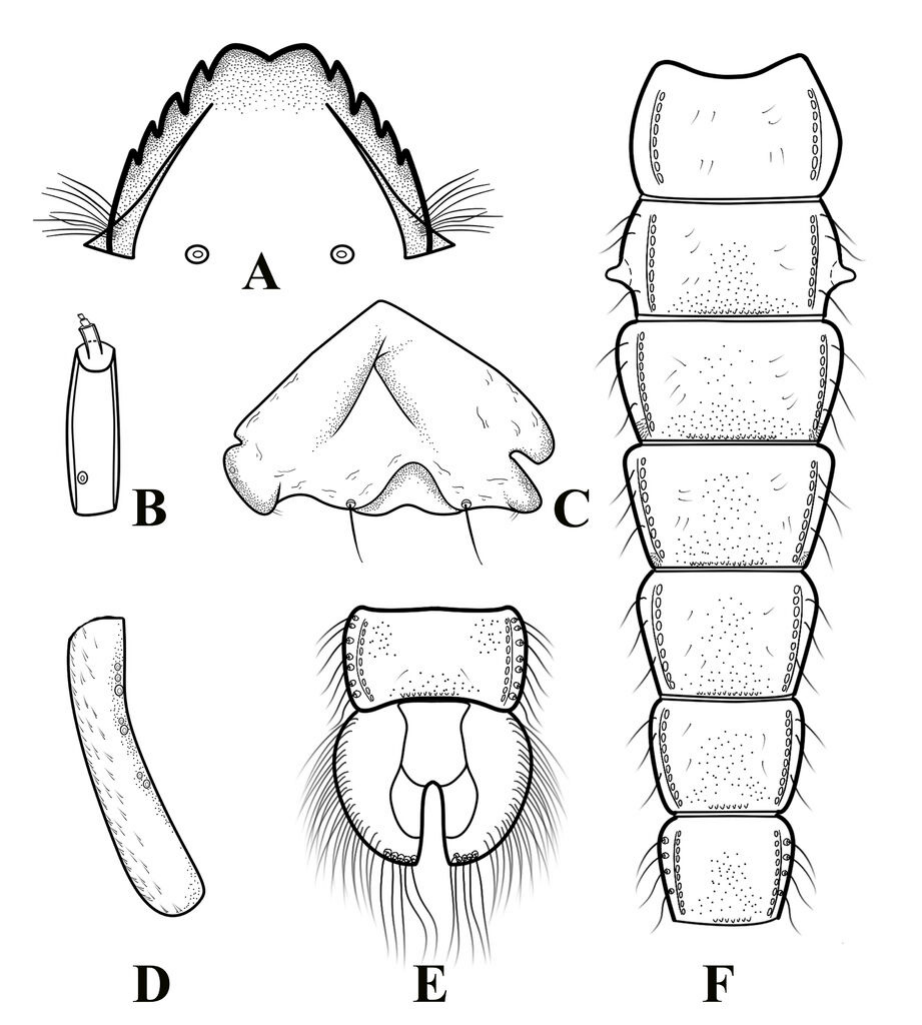
*Psectrocladiusnevalis* Akhrorov, 1977. **A** mentum; **B** antenna; **C** frontal apotome; **D** thoracic horn; **E** sternites VIII–IX; **F** segment I-VII.

Cephalothorax (Fig. 3C-D, Fig. 4C-D). Cephalic tubercles present, frontal setae 58 μm long. Thoracic horn elongate, 290.50 μm long, 42.10 μm wide. Thorax smooth, free of tubercles. Wing sheath without protrusions. Next to the thoracic horn, three precorneal setae are present, lengths of precorneals (µm): 60.20, 125.15, 135.00. 4 dorsocentral setae, the first three are almost evenly spaced, while the fourth one is slightly further away, lengths of dorsocentrals (µm): 50.20, 60.10, 75.15, 76.20.

Abdomen (Fig. 3 A-B, Fig. 4E-F). Sternite I bare; Sternites II–VIII with sparse shagreen; anterior row of anal lobe with shagreen. Sternites II–VIII with posterior row of small spines. Pedes spurii A present in segment III–IV. Pedes spurii B present on segment II. tergites II–VI with 3 Lt-setae; tergite VII with 4 Lt-setae. tergite VIII with 7 Lt-setae. Anal lobe 565.50 µm long and 586.05 µm wide, with 5 subequal anal macrosetae and 20–23 setae in fringe. Anal lobe genital sheath 462.80 µm long, not extending anal lobe.

**Fourth instar larva** (n = 2).

Head capsule (Fig. 3 H) length / width: 525.24– 550.30 µm / 353.45–380.00 µm: 1.45–1.49.

Colouration. The apical 1/3 of mandible, mentum and posterior occipital margin dark brown; the remaining area of head capsule brown.

Antenna (Fig. 3 F and B) with 5 segments, each segment length (μm): 80.31–90.40, 18.25–20.00, 11.42–14.10, 6.50–8.00, 2.10–3.22. Antennal ratio 1.99–2.09. Basal segment almost as long as wide. The distance from base to ring organ 18.87–21.15 µm. Premandible 51.88 μm–60.10 μm long, terminating in a single tooth. Mandible (Fig. 3 G, Fig. 4A) 95.22–101.94 μm long, with 1 apical and 3 inner teeth; apical tooth 24.60–30.72 μm long, nearly equal to the 3 inner teeth in the width. Mentum (Fig. 3 E, Fig. 4A) with 2 median teeth and 4 pairs of lateral teeth, 145.11–156.32 μm wide; a single median tooth almost 2 times larger than the first lateral tooth; postmentum 328.90–350.00 μm long; the distance between the setae submenti 93.75–100.10 μm.

## Discussion

*Orthocladiusnitidoscutellatus* Lundström, 1915.

**Figure 5.**
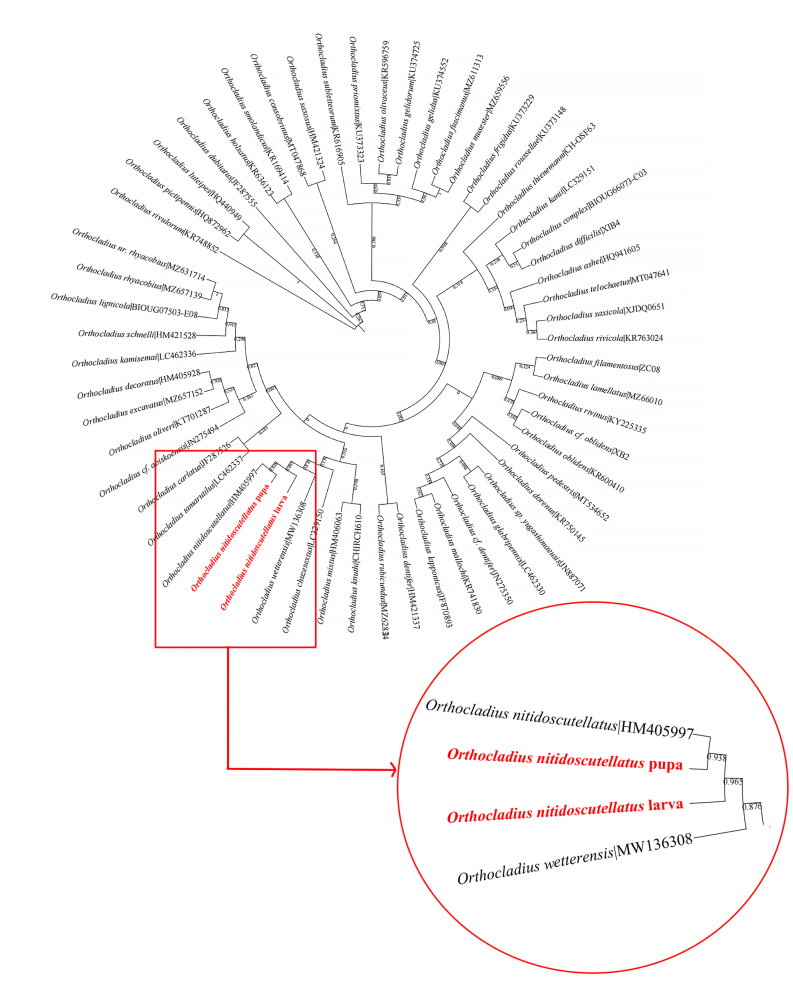
Neighbour-joining tree for known species of the genus *Orthocladius*, based on K2P distances in DNA barcodes. Numbers on branches represent bootstrap support (> 70%) based on 1000 replicates; scale equals the K2P genetic distance.

The larval and pupal specimens were used to extract the COI sequences and compared, all being identified as *Orthocladiusnitidoscutellatus* Lundström, 1915 and these two specimens were well matched. Based on the Neighbour-joining tree of 55 known species in genus *Orthocladius*, the results showed that this species was close to *Orthocladiuswetterensis* Brundin, 1956 in barcode and the two specimens we collected were clustered into one clade with the existing sequences of *O.nitidoscutellatus* Lundström, 1915 (Fig. 5).

*Psectrocladiusnevalis* Akhrorov, 1977.

**Figure 6.**
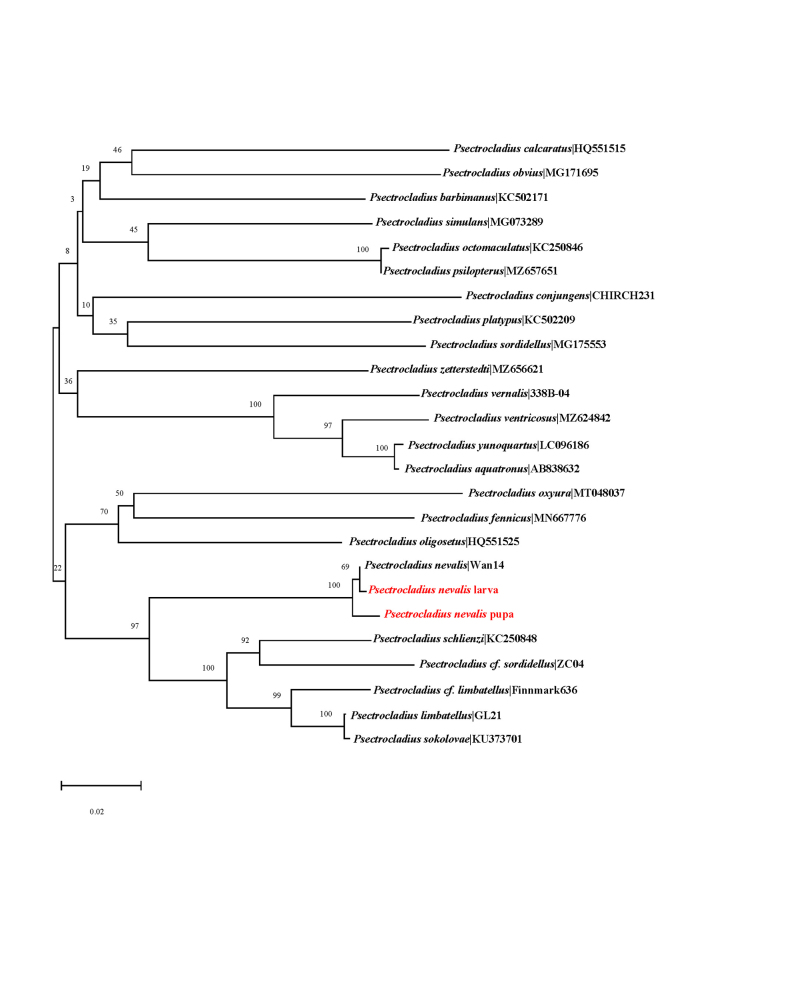
Neighbour-joining tree for known species of the genus *Psectrocladius*, based on K2P distances in DNA barcodes. Numbers on branches represent bootstrap support (> 70%) based on 1000 replicates; scale equals the K2P genetic distance.

The larval and pupal specimens underwent a process of COI sequence extraction and comparison, revealing their identity as *Psectrocladiusnevalis* Akhrorov, 1977. These two specimens demonstrated excellent alignment, further confirming their identity. When plotted on the Neighbour-joining tree of 21 known species within the genus *Psectrocladius*, our collected specimens clustered closely with existing sequences of *P.nevalis* Akhrorov, 1977, as visually represented in Fig. 6.

The molecular identification and morphological taxonomy results align, suggesting that DNA barcodes and traditional morphological taxonomy complement each other, with the former serving as a straightforward means to enhance the latter's effectiveness.

Biogeographically, the two species studied are confined to the Tibetan Plateau, thriving at altitudes exceeding 3,000 m. As altitude rises, the climate becomes increasingly harsh, yet Chironomidae inhabiting these high-altitude regions exhibit remarkable cold tolerance. Simultaneously, this underscores the rich biodiversity of the Tibetan Plateau, indicating a broader distribution range for this genus than previously documented.

In summary, this study not only enhances the Chironomidae database in China, but also contributes vital data towards safeguarding the ecological environment and biodiversity of the Tibetan Plateau.

## Supplementary Material

XML Treatment for Orthocladius nitidoscutellatus

XML Treatment for Psectrocladius nevalis
